# Pancreatic Cancer Malnutrition and Pancreatic Exocrine Insufficiency in the Course of Chemotherapy in Unresectable Pancreatic Cancer

**DOI:** 10.3389/fmed.2020.00495

**Published:** 2020-09-03

**Authors:** Mariia Kiriukova, Daniel de la Iglesia Garcia, Nikola Panic, Maryana Bozhychko, Bartu Avci, Patrick Maisonneuve, Enrique de-Madaria, Gabriele Capurso, Vasile Sandru

**Affiliations:** ^1^Department of Upper Gastrointestinal, Pancreatic, and Biliary Diseases, Moscow Clinical Scientific Center, Moscow, Russia; ^2^Department of Gastroenterology, University Hospital of Santiago de Compostela, Santiago de Compostela, Spain; ^3^Digestive Endoscopy Department, University Clinic “Dr. Dragisa Misovic-Dedinje”, Belgrade, Serbia; ^4^School of Medicine, University of Belgrade, Belgrade, Serbia; ^5^Gastroenterology Department, Alicante University General Hospital, ISABIAL, Alicante, Spain; ^6^Istanbul Faculty of Medicine, Istanbul University, Istanbul, Turkey; ^7^Unit of Clinical Epidemiology, Division of Epidemiology and Biostatistics, IEO European Institute of Oncology, IRCCS, Milan, Italy; ^8^Pancreato-Biliary Endoscopy and Endosonography Division, Pancreas Translational and Clinical Research Centre, IRCCS San Raffaele Scientific Institute, Milan, Italy; ^9^Gastroenterology and Interventional Endoscopy Department, Clinical Emergency Hospital Bucharest, Bucharest, Romania

**Keywords:** pancreatic cancer, locally advanced, metastatic, nutritional status, exocrine pancreatic insufficiency, chemotherapy, dose-intensity

## Abstract

**Background:** Malnutrition and cachexia are common in patients with advanced pancreatic ductal adenocarcinoma (PDAC) and have a significant influence on the tolerance and response to treatments. If timely identified, malnourished PDAC patients could be treated to increase their capacity to complete the planned treatments and, therefore, possibly, improve their efficacy.

**Aims:** The aim of this study is to assess the impact of nutritional status, pancreatic exocrine insufficiency (PEI), and other clinical factors on patient outcomes in patients with advanced PDAC.

**Methods:** PAncreatic Cancer MAlnutrition and Pancreatic Exocrine INsufficiency in the Course of Chemotherapy in Unresectable Pancreatic Cancer (PAC-MAIN) is an international multicenter prospective observational cohort study. The nutritional status will be determined by means of Mini-Nutritional Assessment score and laboratory blood tests. PEI will be defined by reduced fecal elastase levels. Main outcome: adherence to planned chemotherapy in the first 12 weeks following the diagnosis, according to patients' baseline nutritional status and quantified and reported as “percent of standard chemotherapy dose delivered.” Secondary outcomes: rate of chemotherapy-related toxicity, progression-free survival, survival at 6 months, overall survival, quality of life, and the number of hospitalizations. Analysis: chemotherapy dosing over the first 12 weeks of therapy (i.e., percent of chemotherapy received in the first 12 weeks, as defined above) will be compared between well-nourished and malnourished patients. Sample size: based on an expected percentage of chemotherapy delivered of 70% in well-nourished patients, with a type I error of 0.05 and a type II error of 0.20, a sample size of 93 patients per group will be required in case of a percentage difference of chemotherapy delivered of 20% between well-nourished and malnourished patients, 163 patients per group in case of a difference of 15% between the groups, and 356 patients per group in case of a 10% difference. Centers from Russia, Romania, Turkey, Spain, Serbia, and Italy will participate in the study upon Local Ethics Committee approval.

**Discussion:** PAC-MAIN will provide insights into the role of malnutrition and PEI in the outcomes of PDAC. The study protocol was registered at clinicaltrials.gov as NCT04112836.

## Background

Pancreatic ductal adenocarcinoma (PDAC) represents one of the most lethal malignancies nowadays (1), with an age-standardized incidence rate of 4.8 and age-standardized mortality of 4.4 per 100,000 people worldwide. PDAC accounts for the majority of cases of pancreatic neoplasms (2). Diagnostic and therapeutic advancements led to a decrease in mortality of the most cancer types during the last decades; however, PDAC mortality still almost equals the incidence (1), with 5-year survival lower than 10% in the United States (3). The fact that most PDAC patients remain asymptomatic until advanced stages of the disease, as well as the aggressive biological behavior of this tumor and the absence of an effective screening method, largely contribute to these results. However, taking into account that more than 450,000 persons each year are diagnosed with PDAC (1) representing significant healthcare and socioeconomic burden, there is a substantial need for improvement in the diagnostics and therapy in order to prolong survival.

A profound weight loss is one of the early symptoms of PDAC that can precede the diagnosis by months (4). Cachexia, as a symptom and consequence of the disease course, is present in many cancer types, especially in the late stages. Fearon et al. (5) described it as a multifactorial syndrome defined by an ongoing loss of skeletal muscle mass (with or without loss of fat mass) that cannot be completely reversed by conventional nutritional support and leads to progressive functional impairment. In addition to changes such as skeletal muscle wasting or loss of adipose tissue, cachexia is associated with changes in numerous nutritional parameters (6). Among the mechanisms determining the tumor-induced cachexia, the breakdown of molecules due to increased catabolism and inflammation are considered very important (7). Moreover, it has been recently reported that altered pancreatic exocrine function can additionally contribute to cachexia in pancreatic cancer by driving adipose tissue wasting (8).

Cachexia and malnutrition not only represent symptoms of the disease but also are important factors having a significant impact on the outcome of PDAC patients (9). A low value of fecal elastase-1, depicting impaired exocrine pancreatic function and contributing to cachexia, has been reported to strongly correlate with poor survival in advanced PDAC patients (10). However, cachexia and impaired nutritional status might also affect the tolerance and response to medical treatments such as chemotherapy. This is particularly important in PDAC; not more than 20% of patients are eligible for the resection at the time of the diagnosis (3), while the rest might have an indication for chemotherapy. In this setting, while Gemcitabine mono-chemotherapy has for a long time been the standard treatment for unresectable PDAC with relatively poor effect represented by a median overall survival (OS) around 6 months (11), intensified regimens, such as the combination of 5-fluorouracil, leucovorin, irinotecan, and oxaliplatin (FOLFIRINOX), or the addition of Nab-Paclitaxel to gemcitabine, are nowadays more frequently used, with substantially improved survival (12–17). However, due to their side effects, particularly encountered in FOLFIRINOX, intensified regimens can be less tolerable, leading to dose reduction or therapy discontinuation (13–15), which makes their use limited to the patients with ECOG status of 0, 1, and 2 (15, 16). For patients with ECOG of 3 or higher, the large majority of experts recommend best supportive care (18).

Considering this, it is even more important to assess the impact of the nutritional status in patients with advanced PDAC in order to identify sub-groups at a higher risk of side effects and treatment discontinuation. If timely identified, these patients could be approached and treated accordingly, in order to increase their capacity to complete the planned therapy and therefore possibly improve its efficacy. Studies so far conducted to address this important issue have been too few and limited in terms of parameters evaluated as well as outcomes observed and often did not use standardized measures. Hence, evidence is sparse and heterogeneous.

We, therefore, designed a multicenter prospective study in order to assess the impact of patient's nutritional status on the clinical course of advanced PDAC patients receiving chemotherapy.

### Hypothesis

We hypothesize that malnutrition has an adverse impact on the clinical course of patients with advanced PDAC treated with chemotherapy.

### Aims

To investigate the association between the nutritional status and pancreatic exocrine function and the clinical outcomes of patients with advanced PDAC.

## Methods

### Study Design

The PAncreatic Cancer MAlnutrition and exocrine pancreatic INsufficiency in the course of chemotherapy in unresectable pancreatic cancer (PAC-MAIN) study is a non-profit, international, multicenter, prospective, observational, cohort study evaluating the effect of the nutritional status and pancreatic exocrine function on the main outcomes of patients with advanced PDAC. The study will be carried out in Russia, Turkey, Serbia, Romania, Italy, and Spain as a part of the Pancreas 2000 Educational Program. Pancreas 2000 is a post-graduate educational program that prepares young gastroenterologists, surgeons, radiologists, and other physicians for specialization in Pancreatology (19).

#### Patients

Population: patients with unresectable locally advanced or metastatic PDAC attended in each participant's center.

Inclusion criteria:

- age ≥ 18 years;
- histological diagnosis of PDAC within 1 month from recruitment to the study;
- radiological diagnosis of the advanced stage not suitable for upfront surgical resection (either locally advanced or metastatic) within 5 weeks from recruitment to the study (20);
- a written consent to participate in the study;
- being planned for chemotherapy.

The following exclusion criteria will be applied:

- poor performance status (Eastern Cooperative Oncology Group scale (ECOG) ≥ 3) (18)^1^;
- pregnancy;
- past history of any anticancer treatment (surgery and/or chemotherapy);
- enteral nutrition.

#### Variables

The following variables will be recorded in a dedicated Case Report Form (CRF).

All these measures are part of a standard workup of advanced PDAC patients and considered good clinical practice.

Patient-related:
  - sex, race, age at diagnosis
  - significant comorbidities: chronic kidney failure, chronic heart failure, or respiratory insufficiency requiring oxygen treatment
  - Mini-Nutritional Assessment (MNA) score. Primarily developed for elderly patients, MNA score was successfully used in the PreMiO study (Prevalence of malnutrition in patients at first medical oncology visit) to identify the risk of malnutrition or malnutrition among cancer patients at their first medical oncology visit (21):
    ◾ 0–7 points: Malnourished
    ◾ 8–11 points: At risk of malnutrition
    ◾ 12–14 points: Normal nutritional status
  - sarcopenia [measured with computed tomography (CT); fat-free mass is reduced; i.e., appendicular/L2 skeletal muscle mass index < 7.2 kg/m^2^ (men) or <5.5 kg/m^2^ (women)];
  - cachexia [weight loss (WL) > 5% in the last 6 months, or WL > 2% if body mass index (BMI) < 20 kg/m^2^ or sarcopenia];
  - 12-item functional assessment of anorexia/cachexia therapy anorexia/cachexia subscale (FAACT-A/CS-12)
  - a biliary stent
  - a duodenal stent
  - total and direct bilirubin
  - ECOG status
  - European Organization for Research and Treatment of Cancer (EORTC) QLQ-PAN26 scale (22)
  - Date of diagnosis, visit 1, visit 2 (3 months), and death/loss from follow-up
  - Check up on survival at 6 m

Tumor-related:
  - Tumor site documented by endoscopic ultrasound, CT, or magnetic resonance imaging (head, body, or tail)
  - Stage according to the TNM classification
  - Vessels involved
  - Presence and site of metastatic disease
  - Ascites
  - CA-19-9
  - Response evaluation criteria in solid tumors (RECIST) (23) (for visit 2)

Nutritional parameters:
  - Leucocytes (lymphocytes, neutrophils), neutrophil-to-lymphocyte ratio, erythrocytes, hemoglobin, hematocrit, platelets
  - C-reactive protein, total protein, albumin, cholesterol, iron, transferrin, ferritin, magnesium, zinc
  - International normalized ratio, activated partial thromboplastin time
  - Blood fasting glucose, glycated hemoglobin

Pancreatic function and treatment:
  - PEI will be defined by levels of fecal elastase-1 <200 mcg/g; pancreatic enzyme replacement therapy (PERT), date of starting PERT, the dosage of daily taken PERT
  - Diabetes mellitus (DM), date of DM diagnosis, DM type, DM treatment

Treatment-related:
  - Planned chemotherapy protocol
  - Dosages of chemotherapy planned (mg/m^2^)
  - Percent of standard chemotherapy dose delivered
  - Percent of planned chemotherapy delivered
  - Changes to the predefined schedule (dose reduction, schedule modifications, stop before planned)
  - Date of treatment start and end
  - Adverse events (National Cancer Institute toxicity scale for visit 2) (24).

#### Study Period

Depending on approval of the Local Ethics Committees, enrollment is planned to start from March 2019 and last until June 2020 or until the planned power calculation has been met.

#### Outcomes

The association between all the abovementioned variables and the following outcome variables will be assessed:

Primary outcome:

Adherence to planned chemotherapy in the first 12 weeks after the diagnosis in patients' groups stratified according to their baseline nutritional status.

Drug doses will be expressed in weight-based, body surface area (BSA)-based, AUC units or flat dose, according to standard dosing practice for a given drug or combination. For each drug in a regimen, the sum of the doses delivered during the first 12 weeks of therapy will be divided by the sum of the expected doses based on published standard schedule and dosing. The mean percent dose delivered of all drugs in a regimen will be reported as “percent of standard chemotherapy dose delivered.”

Similarly, the sum of the doses delivered during the first 12 weeks of therapy will be divided by the sum of the expected doses based on each patient's starting chemotherapy dose, and the mean percent dose delivered for all drugs in a regimen will be reported as “percent of planned chemotherapy delivered.” We will use percent of standard chemotherapy dose delivered to estimate the overall relative dose delivered, and we will use percent of planned chemotherapy dose delivered to quantify further dose reductions from starting dose and as an indicator of overall toxicity.

Secondary outcomes:

- Percent of patients with chemotherapy-related toxicity in each group of patients
- OS and survival at 6 months
- Progression-free survival
- Quality of life that will be assessed using the European Organization for Research and Treatment of Cancer (EORTC) QLQ-PAN26 scale
- Number of hospitalizations
- Factors associated with the percent of chemotherapy received.

#### Description of the Intervention (Schedule of Visits)

Visit 1 (screening, within 1 month from initial diagnosis)

Patients will be informed about the study. Once patients agree with the inclusion in the study, we will evaluate the inclusion and exclusion criteria. Those patients who meet all the inclusion criteria and none of the exclusion criteria will be finally included in the study. In this visit, patient-related, tumor-related, and pancreatic function and treatment-related variables will be recorded, and quality of life questionnaire will be administered. The researcher will record weight, height, BMI, and unplanned WL % for the last 6 months.

Each patient's baseline nutrition status will be evaluated using the MNA score prior to starting chemotherapy. Patients will be classified as in the group with no nutritional risk, at risk of malnutrition, or malnourished.

Nutritional parameters and pancreatic function will be evaluated through blood tests and a fecal test.

Visit 2 (3 months after the first dose of planned chemotherapy)

The researcher will record in the CRF the planned chemotherapy, schedule, doses, dose reduction, and any adverse event^1^. The same variables recorded at Visit 1 will be checked again.

Check-up 3 (end of the study, 6 months)

The researcher will record in the CRF the OS and time until progression.

#### Medication of the Study

The study is of observational nature, so a pre-planned treatment is not considered. However, the use of pancreatic enzyme replacement treatment will be recorded as well as data regarding the employed chemotherapy regimen.

#### Statistical Analysis

The STROBE guidelines for observational studies will be followed to report our findings^2^. Descriptive statistics (including mean, standard deviation, median, range, frequency, and percent) will be calculated to characterize the study cohort.

Chemotherapy dosing over the first 12 weeks of therapy (i.e., percent chemotherapy received in the first 12 weeks, as defined above) will be compared between well-nourished and malnourished patients. Two-sample *t*-tests/Wilcoxon rank-sum tests will be used for MNA comparisons of mean/median percent chemotherapy received, for all patients and stratified by risk for toxicity. Two-sample *t* tests/Wilcoxon rank-sum tests and chi-square tests/Fisher's exact tests will be used, as appropriate, to compare malnutrition status between groups based on MNA, on demographic/clinical characteristics of interest.

Multivariable logistic regression analysis will be used to estimate the independent effect of malnutrition status on percent chemotherapy received (<80% chemotherapy received vs. 80% chemotherapy received; binary end point), after controlling for demographic and clinical characteristics (age, sex, race, ECOG status, tumor site, tumor stage, PEI, and DM). The demographic and clinical variables included in the final model will be chosen using a forward-stepwise method. Similarly, multivariable linear regression analysis will be used to estimate the independent effect of nutritional status on mean percent chemotherapy received (i.e., continuous endpoint), after controlling for demographic and clinical characteristics. All *p* values are two-sided with statistical significance evaluated at the 0.05 alpha level. Ninety-five percent confidence intervals (CIs) will be calculated to assess the precision of the obtained estimates (for odds ratios/beta estimates). The Kaplan–Meier method will be used to estimate OS and progression-free survival (PFS). The log-rank test will be used to compare OS and PFS between well-nourished patients and malnourished patients. Greenwood's formula will be used to calculate 95% CIs for Kaplan–Meier survival estimates. The frequency and percentage of missing values for each variable will be collected, analyzed, and reported (missing value analysis). All data will be anonymous once data collection is completed, respecting the confidentiality of the subjects participating, in accordance with data protection laws. All analyses will be performed in STATA 14 (Statacorp LLC, Texas).

#### Power Size Calculation

The expected percent of chemotherapy delivered in well-nourished patients was based on a study that assessed the chemotherapy dose intensity in gastrointestinal malignancies that included pancreaticobiliary disease during the first 8 weeks after the start of the chemotherapy (25). Based on an expected percentage of chemotherapy delivered of 70% in well-nourished patients, with a type I error of 0.05 and a type II error of 0.20, a sample size of 93 patients per group will be required in case of a percentage difference of chemotherapy delivered of 20% between well-nourished and malnourished patients, 163 patients per group in case of a difference of 15% between both groups, and 356 patients per group in case of 10% difference.

#### Ethics, Registration, and Dissemination

The study will be performed in accordance with the Declaration of Helsinki (2013) as well as the Good Clinical Practice International Ethical and Scientific Quality Standards. The study protocol was approved by the Local Ethics Committee in the leading center of the study, A.S. Loginov Moscow Clinical Scientific Center in Moscow, Russia (extract No 2/2019) on the 18th of February 2019 and by local Institutional Review Boards of all participating/collaborating centers. The database will not contain names or identification numbers that may compromise patient anonymity and will be stored online using REDCap with secured (username and password) and limited access only to the members of the study group mentioned above and in accordance with The General Data Protection Regulation of the European Union. Participation in the study will be voluntary, after signed informed consent. The written informed consent will be obtained by the study collaborators in each participating center. The study protocol was registered at clinicaltrials.gov as *NCT04112836*. The results of the study will be disseminated among representatives of the medical community through dedicated medical conferences and published articles.

## Discussion

Given the sparse overall scientific data on the subject, we have designed a study that addresses the impact of nutritional status and dietary intervention on the clinical course of patients with advanced PDAC treated with chemotherapy and aims to establish whether it affects both tolerance and tumor response to medical therapy. PAC-MAIN will be the first targeted study for investigating whether the nutritional status influences the possibility to complete planned chemotherapy in patients with advanced PDAC.

## Author Contributions

MK, DI, NP, MB, BA, PM, Ed-M, GC, and VS participated in designing the protocol and in drafting the manuscript. DI and PM provided statistical expertise in clinical trial design. All authors contributed to the refinement of the study protocol and approved the final manuscript.

## Conflict of Interest

The authors declare that the research was conducted in the absence of any commercial or financial relationships that could be construed as a potential conflict of interest.

## Footnotes

^1^https://ecog-acrin.org/resources/ecog-performance-status

^2^https://strobe-statement.org
